# Magnetic Simulations of Core–Shell Ferromagnetic Bi-Magnetic Nanoparticles: The Influence of Antiferromagnetic Interfacial Exchange

**DOI:** 10.3390/nano11061381

**Published:** 2021-05-24

**Authors:** Juan A. Ramos-Guivar, Carlo A. Tamanaha-Vegas, Fred Jochen Litterst, Edson C. Passamani

**Affiliations:** 1 Grupo de Investigación de Nanotecnología Aplicada para Biorremediación Ambiental, Energía, Biomedicina y Agricultura (NANOTECH), Facultad de Ciencias Físicas, Universidad Nacional Mayor de San Marcos, Av. Venezuela Cdra 34 S/N, Ciudad Universitaria, Lima 15081, Peru; carlo.levano@unmsm.edu.pe; 2 Institut für Physik der Kondensierten Materie, Technische Universität Braunschweig, 38106 Braunschweig, Germany; j.litterst@tu-braunschweig.de; 3 Centro Brasileiro de Pesquisas Físicas, Rio de Janeiro 22290-180, Brazil; 4 Department of Physics, Federal University of Espirito Santo-UFES, Vitória 29075-910, Brazil; edson.caetano@ufes.br

**Keywords:** atomistic simulation, core–shell bi-magnetic nanoparticles, Monte Carlo simulation, interfacial exchange

## Abstract

Magnetic properties of ferromagnetic nanostructures were studied by atomistic simulations following Monte Carlo and Landau–Lifshitz–Gilbert approaches. First, we investigated the influence of particle size and shape on the temperature dependence of magnetization for single cobalt and gadolinium nanoparticles and also in bi-magnetic Co@Gd core–shell nanoparticles with different sizes. The Landau–Lifshitz–Gilbert approach was subsequently applied for inspecting the magnetic hysteresis behavior of 2 and 4 nm Co@Gd core–shell nanoparticles with negative, positive, and zero values of interfacial magnetic exchange. We were able to demonstrate the influence of finite-size effect on the dependence of the Curie temperature of Co and Gd nanoparticles. In the Co@Gd core–shell framework, it was possible to handle the critical temperature of the hybrid system by adjusting the Co core size. In addition, we found an improvement in the coercive field values for a negative interfacial exchange energy and for a different core size, suggesting an exchange spring behavior, while positive and zero values of interfacial exchange constant showed no strong influence on the hysteresis behavior.

## 1. Introduction

The thermal dependence of the magnetization, *M(T)*, of low dimensional ferromagnets (FM) has been studied and simulated by different methods using the Hinzke–Nowak Monte Carlo (MC) algorithm [1], the magnetic dynamic Landau–Lifshitz–Gilbert (LLG) equation [2], and very recently by the Heisenberg mean-field theory [3]. The Curie temperature (*T_C_*) of FM in the nanoscale regime is still an open issue. It is basically due to the fact that the *T_C_* value will depend on the finite-size effects, when long-range atomic ordering is lost due to material sizes and surface morphologies [4].

A magnetic material with a low *T_C_* value (near to room temperature) is often required for applications, for instance, in magnetic hyperthermia [5]. Some good examples of these materials are doped nanoparticulate systems, such as La_1–x_Sr_x_MnO_3_ [6], Mn_1–x_Zn_x_Fe_2_O_4_ nanoparticles (NPs) [7], and hard–soft mixed ferrites [5]. In addition to this peculiarity, these magnetic nanosystems can be coated with gadolinium (a rare earth metal with a *T_C_* of 293 K that is relatively low as compared to ordinary 3D-FM [8]) to be used as a contrast agent in Magnetic Resonance Imaging (MRI) recording [9,10]. Thus, the understanding of the magnetic interactions and the *M(T)* behavior, which govern the core–shell magnetic nanosystems, are crucial, at least for future biomedical applications. In fact, the *M(T)* behavior in bi-magnetic NPs has been theoretically and experimentally studied in some systems [11,12] but not in cobalt and gadolinium (Co@Gd) core–shell structures having different geometries. 

On the other hand, several models have theoretically been employed to study the superparamagnetism in ensembles of magnetically non-interacting and interacting NPs. For instance, Serantes et al. [13] simulated the behavior of an ensemble of non-interacting identical particles contained in a 3D box. The implementation of the effect of superparamagnetism was done by taking into account the classical formulation of Stoner–Wohlfarth using the MC method. Another formulation was considered by Arantes et al. [14], who implemented the classical Heisenberg model also for non-interacting frozen magnetic particles. They traced the transition from the magnetically blocked to the superparamagnetic regime using the concept of blocking temperature that showed an intrinsic dependency with the finite-size effect. Obviously, when the system's complexity is enhanced for nanostructures (including different magnetic exchange types), the simulation times get longer (i.e., costlier), and therefore, new simulation methods need to be developed and implemented to analyze the temperature dependence of magnetic properties of core–shell-like nanostructures. Especially for a combination of magnetically hard and soft magnets, one may expect a magnetic exchange interaction between these two FM phases, resulting in an exchange spring (ES)-like phenomenon. Thus, the hybrid materials will have enhanced magnetic properties such as high coercivity and also high saturation magnetization, as reported by Coehoorn et al. [15].

Considering the above points, we simulated magnetic properties of single Co and Gd NPs and also the nanohybrid Co@Gd core–shell structure by atomistic simulations using VAMPIRE software. We performed several studies applying the MC method in magnetic nanosystems for the following cases: (i) the *M(T)* behavior of individual FM Co and Gd NPs with different geometries (spherical, cubic, and cylindrical) in the range from 1 to 9 nm, (ii) core–shell spherical systems of Co@Gd, where the thermal dependence of the critical temperature was studied using different interfacial exchange values with an insignificant change in the *T_C_* values, suggesting that the bi-magnetic core–shell system can be employed in biomedical applications for imaging contrast with high magnetic response, and finally (iii) the hysteretic behavior of the Co@Gd core–shell system was studied by changing the Co core size and the interfacial exchange constant. An improvement in the coercive (*H_C_*) field was observed for a negative interfacial exchange, suggesting the existence of the ES effect. Thus, from our results it is possible to significantly increase the coercive values and handle the presence of ES behavior at the core–shell bi-magnetic interfaces.

## 2. Theoretical Background, Models, and Methodology

### Atomistic Simulations Using VAMPIRE

In the framework of the atomistic system generation, VAMPIRE 5.0 software (York, England) [2] was used to generate particles with certain geometrical shapes (spheres, cylinders, and cubes). The first step was to create a crystalline network with a size large enough to contain the simulated shape. The exchange interaction of the unit cell is known if one considers the first neighbors’ approach, given by Equation (1). Once the unit cell is replicated until the dimensions agree with the first step, atoms are removed from the crystalline structure until a desired geometrical shape is reached [2]. The atoms within this already molded crystalline structure are then assigned to one or more materials. This also enables the formation of core–shell structures, but it will depend on the core structure, shell, and interfacial exchange interactions.

Two protocols were established for simulations with the VAMPIRE software:

(i) To reach equilibrium for a subsequent study of the *M(T)* properties, a Hinzke–Nowak MC algorithm was employed, using 20,000 equilibrium steps. After 20,000 averaging steps, the system was then heated in steps of 5 K till the complete *M(T)* curve was obtained. To investigate the finite-size effects on *T_C_*, the equilibrium magnetization for different sizes of spherical, cylindrical, and cubic NPs were calculated as a function of temperature, *M(T)*. The Hamiltonian for the simulated system is [2]:

(1) $$H=-\underset{i\neq j}{\sum }J_{ij}S_{i}.S_{j}$$

where $J_{ij}$ is the FM exchange constant, and *i,j*, refer to FM sites. For the simulations, we fixed *J_ij_* = 6.064 × 10^−21^ J/link (=joule/binding) and Co atomic spin moment of 1.72 μ_B_ (=bulk value of moment). In the case of Gd, *J_ij_* = 1.28 × 10^–21^ J/link and Gd atomic spin moment of 7.63 μ_B_ (bulk value). The exchange integral (J) is defined, based on mean field theory, as [2]

(2) $$J=\frac{3k_{B}T_{c}}{\epsilon z}$$

where $k_{B}$ is the Boltzmann constant, *z* is the number of nearest neighbors, and ϵ is a correction factor obtained from the mean-field expression [2].

**Figure 1:**
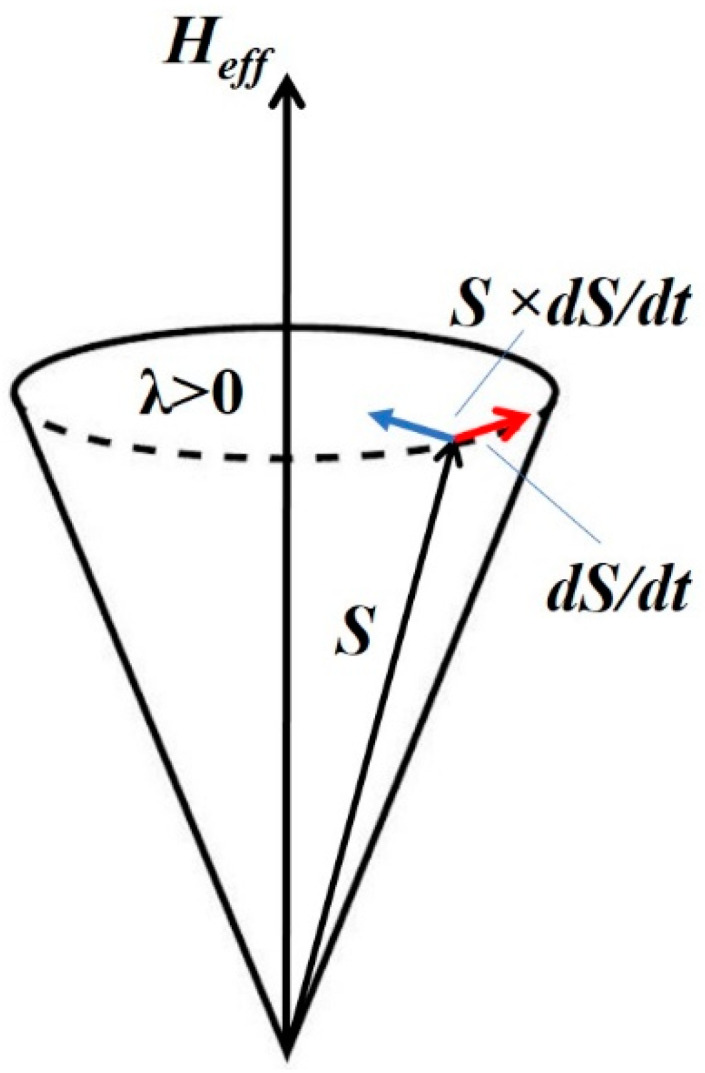
Sketch of spin precession.

For the core–shell model (e.g., negative interfacial configuration), the Hamiltonian Equation (1) turns to

(3) $$H_{exc}=H_{FM1}+H_{AFM}+H_{FM2}=\sum_{i\neq j}J_{FM1}S_{i}.S_{j}+\sum_{i,v}J_{afm}S_{i}.S_{v}+\sum_{v,i}J_{afm}S_{v}.S_{i}+\sum_{i\neq j}J_{FM2}S_{i}.S_{j}$$

where *S* corresponds to the spin unit vector. In Equation (3), the first term refers to FM Co, the last one to the shell of Gd, and the second and third terms are the interfacial exchange energies that, in the VAMPIRE software, are taken with the same value. In other words, Equation (3) describes only exchange coupling between the two FM structures, and other interactions (dipole–dipole, anisotropy, and Zeeman, e.g.) to the total magnetic energy are considered negligible. The dipolar was discarded because we assumed non-interacting NPs, while the magnetic anisotropy was due to the fact that the temperature-dependent magnetic properties of the nanohybrid Co@Gd core–shell system were mainly associated with the strong relation between *T_C_* and *J* given by Equation (2). The Zeeman contribution was neglected since it is still not implemented in the VAMPIRE software using the MC approach. We only took into consideration the interfacial exchange and direct exchange in the FM core and shell, respectively. In brief, the above model assumes the fact that NPs are monodisperse, and the effect of magnetic interactions is expected to be negligible.

(ii) The Landau–Lifshitz–Gilbert (LLG)–Heun method, implemented in the software VAMPIRE [2], was used to simulate the hysteresis curves [*M(H)*], using Equation (4) as suggested in Ref. [2]:

(4) $$\frac{\partial S_{i}}{\partial t}=-\frac{\gamma }{\left(1+\lambda^{2}\right)}\left[S_{i}\times H_{eff}^{i}+\lambda S_{i}\times \left(S_{i}\times H_{eff}^{i}\right)\right]$$

where *S_i_* is the unit vector that represents the direction of the magnetic spin moment *i*, *λ* is the microscopic damping parameter, *γ* is the gyromagnetic ratio, and $H_{eff}^{i}$ is the net effective magnetic field on each spin [2] (see Figure 1 for a sketch of the spin precession model). In Equation (4), the $H_{eff}^{i}$ is a sum of two contribution as given by Equation (5):

(5) $$H_{eff}^{i}=-\frac{1}{\mu_{S}}\frac{\partial H}{\partial S_{i}}+H_{th}^{i}$$

where the first term is the Hamiltonian used for the LLG method as represented by Equation (6), and the second term is the thermal field, given by Equation (7), as follows:

(6) $$H=H_{exc}+H_{ani}^{uni}+H_{app}$$

(7) $$H_{th}^{i}=\Gamma \left(t\right)\sqrt{\frac{2\lambda k_{B}T}{\gamma \mu_{S}\Delta t}}$$

where $H_{ani}^{uni}$ is the uniaxial magnetic anisotropy and external applied magnetic field contribution, *Γ(t)* is a Gaussian distribution that represents the thermal fluctuations of each spin *i*, and $\mu_{S}$ is the magnitude of the atomic magnetic moment.

For core–shell models, the same input and mat files used for *M(T)* curves were used as templates, but the magnetic field was varied from −3T to +3T with an applied field strength increment of 0.01 T. The λ value was taken equal to 1.0 for an elemental FM as implemented by [2] for all simulations, and the same Hamiltonian given in Equation (4) was used. Additionally, it is important to note that all magnetization data were presented as normalized magnetization (*M/M_s_*), where *M_S_* is the saturation magnetization. For all simulations performed in this work, a simple laptop HP Intel Core i3, serial number 5CB4211908, was used.

## 3. Results and Discussion

We start this section pointing out that for magnetic configurations (e.g., core–shell structures), a single MC program for non-interacting NPs needs to be modified. Otherwise, it will increase the MC step to a much higher value, as those employed by Nehme et al. [16], where up to 100,000 MCs were used to stabilize the physical parameters of a non-interacting system. This, of course, will increase the computation time significantly to some days or even weeks depending on the Random Access Memory (RAM) computer memory and multi-core processor. On the other hand, the VAMPIRE software allows one to perform atomistic simulations in a single graphics processing unit (GPU) for a variety of magnetic configurations using MC and LLG approaches in much shorter computational times. The software runs easily in serial mode with optimized computational time. Based on that, we applied the VAMPIRE software to study FM bi-magnetic core–shell structures, and the results are shown in the next sections.

### 3.1. T_c_ Finite-Size Effects for Single Gd and Co FM NPs with Different Geometries

**Figure 2:**
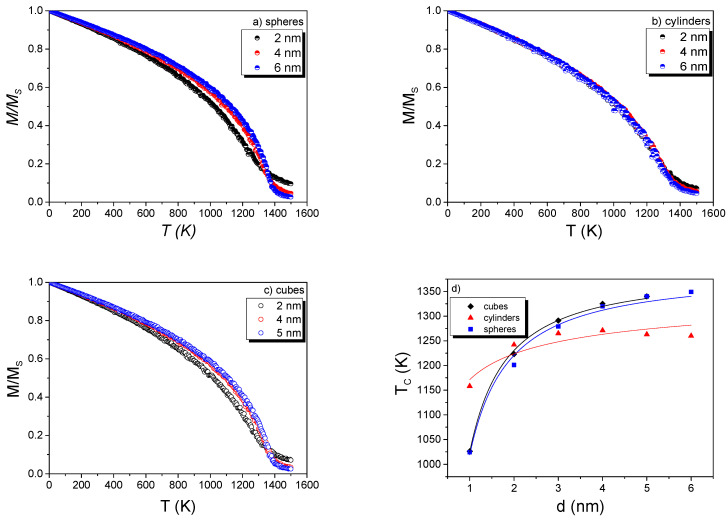
Normalized *M(T)/M_s_* curves for an ensemble of non-interacting Co NPs with sphere (**a**), cylinder (**b**), and cube (**c**) geometries. (**d**) Size dependence of the *T_C_* for the three studied geometries. (in (**d**) the full lines are guides for eyes).

**Figure 3:**
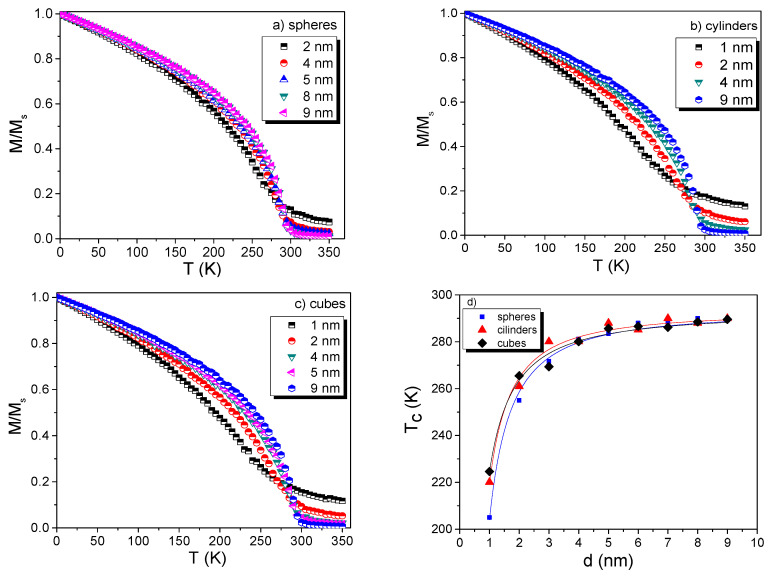
Normalized *M(T)/M_s_* curves for the Gd NPs with sphere (**a**), cylinder (**b**), and cube (**c**) geometries. (**d**) Size dependence of the *T_C_* for the three studied geometries. (in (**d**) the full lines are guides for eyes).

Before studying the core–shell structures, the *M(T)* behavior was systematically studied independently for single Co and Gd nanostructures with three geometries (sphere, cylinder, and cubic). For the spheres, the diameters were in the range from 1 to 9 nm. For the cube geometry, the cubic length for each face was the same in the rectangular (x,y,z) dimensions and increased from 1 to 9 nm, while for the case of cylinder geometry, the side length increased from 1 to 9 nm, and the base diameter of the cylinder was fixed to 2 nm for all simulations. The results are respectively shown in Figure 2 and Figure 3. A clear distinction in the *M/M_S_* (hereafter called as *M(T)*) profiles is observed with decreasing size. For 1 to 6 nm, the *M(T)* curves have a tendency to increase continuously above *T_C_*_,_ as would be expected for small FM NP systems (not behaving as an ordinary FM that would show a Brillouin-like curve).

For the determination of *T_C_*, three approximations were employed [1]: (i) Curie–Bloch (Equation (8)), (ii) Kuzmin relation (Equation (9)), and iii) derivative criteria and subsequent polynomial fitting. A first fitting was performed using the Equations (8) and (9):

(8) $$m\left(T\right)={\left(1-T/T_{c}\right)}^{\beta }$$

(9) $$m\left(\tau \right)={\left[1-s\tau^{\frac{3}{2}}-\left(1-s\right)\tau^{p}\right]}^{\beta }$$

where β is suggested to be a universal exponent equal to 0.34 for ideal FM and $\tau =\frac{T}{T_{C}}$, *s* = 1, and *p* = 3/2 for a pure Bloch FM [1]. However, the Equations (8) and (9) cannot be used to fit the simulated *M(T)* data when particle sizes are below 9 nm. Values of *β* ranging from 0.45 to 0.34 were obtained for sizes in the range of 1 to 9 nm, respectively, and consequently no accurate determination of *T_C_* could be obtained. Hence, the third approximation was employed, and the obtained *T_C_* values vs d(nm) are plotted in Figure 2d and Figure 3d for Co and Gd NPs, respectively. *T_C_* values decreased for individual Co and Gd NPs for sizes smaller than 5 nm. As is well-known from the literature [17], the *T_C_* has a finite-size dependence for which critical parameters can be determined using Equation (10):

(10) $$\in \left(d\right)=\frac{T_{c}^{\infty }-T_{c}\left(d\right)}{T_{c}^{\infty }}={\left(\frac{d_{0}}{d}\right)}^{z}$$

where *z* is the phenomenological shift exponent, *d* (nm) is the length of the NPs geometries (diameters for spheres, and cubic and side cylinder lengths), and *d_0_* is the microscopic length close in value to the single unit cell in the lattice structure of the FM material. 

For smaller particle sizes, the magnetic behavior, near *T_C_*, loses its critical magnetic behavior, and *T_C_* can hardly be determined accurately [2]. Commonly, *T_C_* is taken from the minimum of *dM/dT*. However, at nanoscale, this significantly underestimates the actual temperature in which the magnetic order is lost. We resolved this issue by fitting the *dM/dT* curve with a polynomial fitting that covers the total curve, including all points and not only the minimum value. The estimated values are individually reported in Table S1 for Co and Gd NPs (they are not much different from their bulk counterparts). Another visible effect, for very small NPs, is the slightly different behavior of the magnetization when the temperature is increasing (not a pure Brillouin-like curve as expected for an ordinary FM, i.e., the increasing tendency of magnetization above *T_C_* is different from bulk FM). This effect on the magnetization has been pointed out to occur from the correlations of local moments that are presented above *T_C_* [2,17]. It only plays a role in NPs when the size of the system is close to the length of the magnetic correlation, *ξ* [2,17]. Moreover, the critical exponent for Co NPs (Table S1) is close to the value previously reported for Co films, that is, *z* = 1.34 [18]. In Table S1, there are also shown *z* values for the different geometries. In a first approximation, the z values (Table S1) are similar to that found also by Fisher [19], i.e., *z* = 1. Observing the *T_C_* values (Table S1), one can notice that only the cylinder NPs geometry has shown a change in the *T_C_* value [4], but the values are, in general, not substantially modified when compared among them. In principle, the demagnetization field can hardly change the *T_C_* values of FM described by mean-field theory. Thus, since there were no significant changes in the *T_C_* values upon changing the geometries, especially for spherical geometries in both Co and Gd NPs, we tested the Co@Gd core–shell simulations with spherical-like morphology, because (i) experimentally this is a geometry well accepted to describe real system, (ii) it is a geometry that helps the simulation regarding the spin configuration, and (iii) it is used in the major theoretical, experimental, and application approaches.

### 3.2. Core Shell Model Co@Gd (Effect on Tc Behavior with Size and Interfacial Exchange)

**Figure 4:**
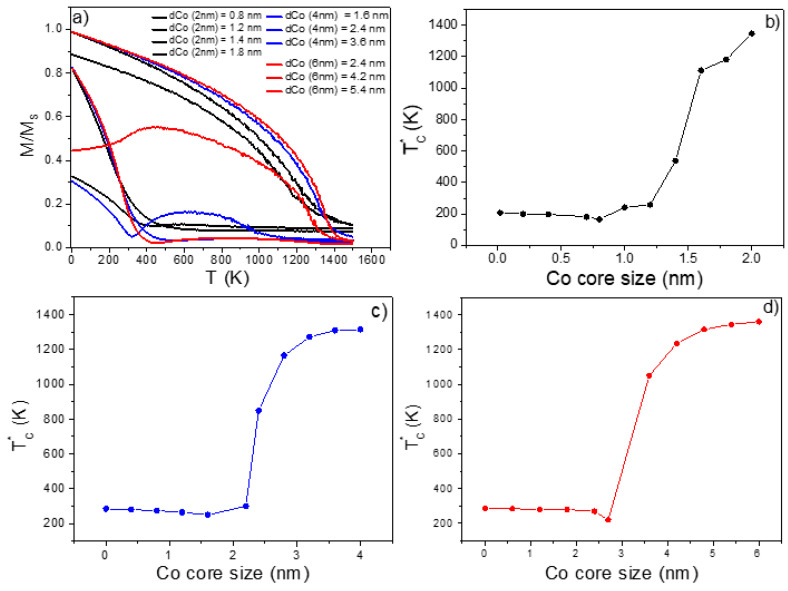
Normalized *M(T)/M_s_* curves for the core–shell bi-magnetic Co@Gd NP with total diameter of 2 (black lines), 4 (blue lines), and 6 nm (red lines) (**a**) for a negative interfacial exchange. Dependence of the $T_{C}^{*}$ on the Co core size for a total particle size: 2 nm (**b**), 4 nm (**c**), and 6 nm (**d**). Full lines in (**b**–**d**) are guides for eyes.

In the present section, different exchange couplings were used to describe the nanohybrid Co@Gd core–shell properties. For J > 0 (parallel spin configuration of the two FM phases), an artificial ferromagnetic-like heterostructure was achieved, whereas for J < 0 (antiparallel spin configuration), the system behaved as an artificial ferrimagnet-like (FI). We then worked with a spherical-like configuration where the Co core size was varied with a total core–shell size of 2, 4, and 6 nm, using values of interfacial exchange of *J_int_* = −1.38 ×10^−21^ J/link This is a value that was taken from the literature for an ensemble of core–shell bi-magnetic NPs [11]. As shown from Figure 4a, the *M(T)* curves have different behaviors, depending on the Co core sizes. Consequently, it has a strong influence in the critical temperature ($T_{C}^{*}$) value of the nanohybrid core–shell system. 

We noticed that for core sizes smaller than 2 nm with total variable size (Figure 4b–d), the $T_{C}^{*}$ values of the Co@Gd core–shell decreased significantly. Especially, it reached a value close to that found for pure spherical Gd NPs (~290 K), as can be seen comparing with data shown in Figure 3a. Thus, this $T_{C}^{*}$ reduction with decreasing of Co size results in a nanohybrid core–shell material with a $T_{C}^{*}$ close to RT (Gd-based materials are shown to present a giant magnetocaloric effect [20,21]).

It is important to highlight the occurrence of a compensation-like temperature in several *M(T)* curves, but not in all of them (see Figure 4a). In principle, this phenomenon only happens due to the temperature dependence of the magnetization of the antiferromagnetically coupled FM Co and Gd sublattices [22,23]. In other words, considering that both sublattices (Gd and Co) have different temperature-dependent behaviors, as the temperature increases, the Gd sublattice will go first to the paramagnetic (PM) state above 293 K, while the Co sublattice will enter into a PM state at higher temperatures. Thus, in a specific temperature of the sample, there will be total magnetization cancellation in case of an ordinary compensation temperature effect. 

However, according to our findings, the compensation-like temperature occurs in all nanohybrid Co@Gd core shell systems independent on the sign of exchange interaction, as also displayed in Figures S1 and S2. Thus, we can point out that this effect should be dependent on the fraction (and magnetic state) of each FM structure that forms the nanohybrid Co@Gd core–shell system (the value of the Gd moment is much higher than that of Co). However, for the nanohybrid Co@Gd core–shell with *J_int_* < 0, one can assume the ordinary compensation temperature effect, and therefore the simulated bi-magnetic core–shell systems behave as a total ferrimagnet with two antiferromagnetically coupled FMs [24]. This interesting effect has been studied in many magnetic systems based on magnetic garnets [22,23] and alloys of ferrimagnetic CoGd [25], but not yet in core–shell Co@Gd NPs. In addition, it can be mentioned that the compensation-like effect physically occurs in a state of high magnetic entropy, consequently favoring the magnetocaloric phenomenon in all nanohybrids here studied, and hence would allow the use of the bi-magnetic core–shell system in biomedical applications. This finding is significantly important since this Co@Gd core–shell system may be applied in magnetic hyperthermia, where systems with *T_C_* close to room temperature (RT) are required.

### 3.3. Hysteresis Curves Simulation 

#### 3.3.1. Determination of the Computational Time Step

To simulate the *M(H)* curves for bimetallic FM core–shell Co@Gd, we made use of Equation (4), using a λ value equal to 1.0 for all the simulations. We first evaluated the suitable computational time step Δ*t*, which represents the numerical parameter employed to resolve the LLG equation with higher precision, as suggested by Aurélio et al. [26]. As can be noticed from Figure S3a, the *M(H)* curves have the expected physical behavior for orders of Δt = 10^−15^ s. In our case, we took a Δ*t* value equal to 2 × 10^−15^ s at 0 K for a spherical 4 nm Co NP. For other orders of Δ*t*, the system does not even reach the saturation regime and no physically reasonable values are obtained, which can be significantly affected when evaluating the *H_C_* field values. Thus, we took a fixed value of 2 × 10^−15^ s for all *M(H)* simulated experiments as taken in [26].

It is important to highlight that this parameter will decide how long the atomistic simulations will take, recalling that the smaller the values, the more expensive will be the computational time and, of course, it will be limited by the particle size (number of atoms). Figure S1b also depicts the Δ*t* dependence for *H_C_* and the *M/M_s_* ratio, where higher values equal to 2 x 10^−15^ s favor the stabilization of both parameters and hence their correct determination.

#### 3.3.2. Hysteresis Curves for Pure 4 nm Co NP

**Figure 5:**
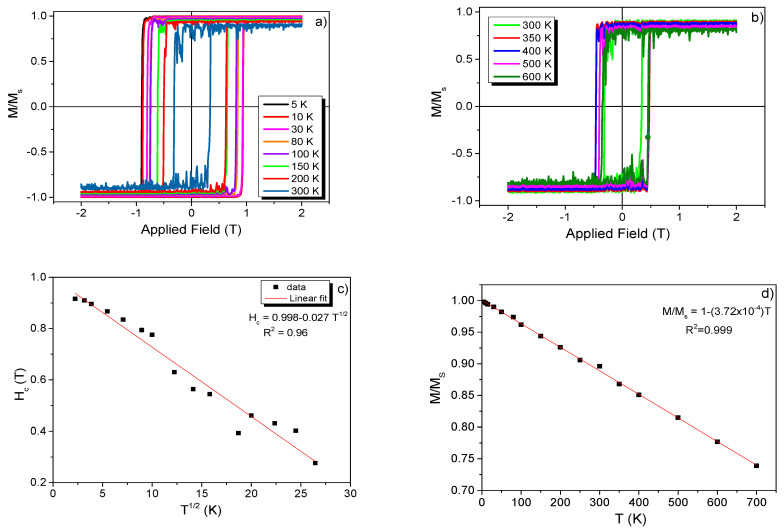
Temperature dependence of the normalized *M(H)/M_s_* curves for the 4 nm Co NPs (**a**,**b**). Thermal dependence of *H_C_* and *M/M_s_* parameters. In (**d**), the ratio *M/M_s_* depends on the temperature and external magnetic field. In (**c**) and in (**d**) the full lines are result from the fits.

Having defined and fixed the Δ*t* values, we first proceed with the estimation of the *H_C_* values for single Co NP at 0 K and several temperatures, as plotted in Figure 5a,b. We would also like to give a reminder that the LLG differential equation is only applicable at 0 K. Therefore, the Langevin dynamics concept must be employed [2] using the *Γ(t)* concept, as represented by Equation (7). Then, to perform the atomistic simulations at other temperatures, the VAMPIRE software used the concept of the thermal field. Figure 5c shows that the *H_C_* field starts to decrease significantly with temperature for simulations up to 700 K, in agreement Kneller’s law [27]. Moreover, the *M/M_s_* linearly with temperature (Figure 5d), and consequently a linear fit with R^2^ equal to 0.999, support this finding.

#### 3.3.3. Hysteresis for Bi-Magnetic Core–Shell NP Varying the Interfacial Exchange Constant

**Figure 6:**
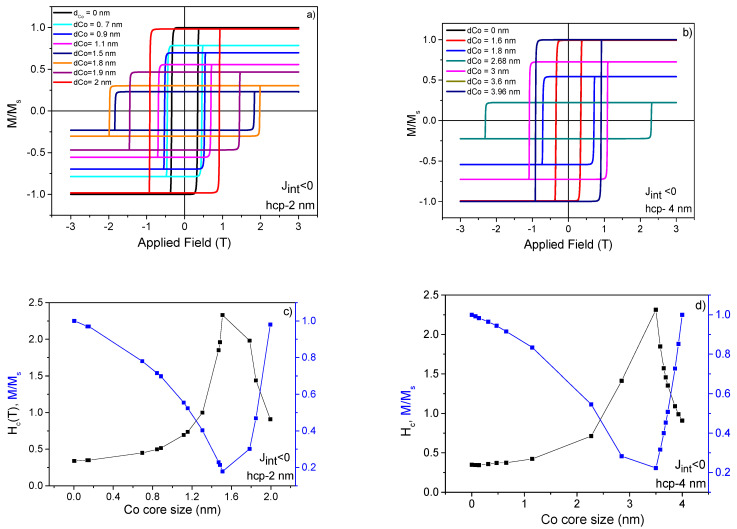
Normalized *M(H)* curves for different Co core size for a total core–shell size of 2 nm (**a**) and 4 nm (**b**) for negative interfacial exchange constant. Dependence of the Co core size of the *Hc* and *M/M_s_* values for 2 nm (**c**) and 4 nm (**d**). In (**c**,**d**), full black and blue lines are guides for eyes.

Sizes of 2 and 4 nm for the bi-magnetic FM Co@Gd core–shell NPs were taken for simulations. The *M(H)* loops for each size were obtained for negative, positive, and zero interfacial exchange (*J_int_*) constants (see Figure 6, Figure S4 and S5). In the case of *J_int_* < 0, the bi-magnetic core–shell NP system exhibited an improvement in the *H_C_* values for both sizes, as can be observed by broad *M(H)* loops displayed in Figure 6c,d. Interestingly, it occurred when the Co core size ranged from 1.2 to 1.5 nm. This means that under the present magnetic configuration (antiparallel spin interfacial exchange) the core–shell system will have an ES-like behavior. This kind of magnetic exchange occurs in systems composed of hard and soft magnetic materials with comparable or small exchange length [28,29]. Therefore, we can assume that the finite-size effect (in this case NP of 2 and 4 nm) will have a strong influence on the appearance of the ES phenomenon. In our system, the magnetic hard core of Co was expected to retain the uniaxial anisotropy of the Gd soft shell, causing an increment of the *H_C_* values. In case of parallel magnetic configuration (*J_int_* > 0), the core–shell *M(H)* loops were not too broad, as seen for *J_int_* < 0, but an enhancement in the *H_C_* values was also observed (Figure S4c,d) in the range from 1.2 to 1.5 nm (for 2 and 4 nm of core–shell size). On the other hand, the *H_C_* value reached 38% of the maximum value (*Hc* = 2.3 T) estimated for the antiparallel interfacial configuration (it became constant with the Co core size). For a zero interfacial exchange configuration, there was no magnetic interaction between the core and the shell. In this case, the magnetic exchange response happened individually for core and shell, and we could clearly observe two superposed magnetic hysteresis loops in the simulated *M(H)*, as displayed in Figure S5a,b. This could also be observed from the Co core diameter dependence of the *H_C_* and *M/M_s_* values that exhibited two independent responses due to Gd and Co, as plotted in Figure S5c,d. Additionally, the *M/M_s_* values were constant for individual non-coupled FM for 2 and 4 nm. This was expected because no temperature effect was taken into account.

## 4. Conclusions

In this work, we first performed atomistic spin model simulations (using free VAMPIRE software) of the temperature-dependent magnetization of Co and Gd single NPs with spherical, cubic, and cylindrical shapes. Regarding the magnetic properties of the FM Co, Gd, and Co@Gd core–shell systems, we have to emphasize that the finite-size effect was observed for all the geometries with exponential critical parameters related to ordinary ferromagnets. We also demonstrated that the $T_{C}^{*}$ value of a core–shell bi-magnetic nanoframework can be tuned by changing the Co core size with spherical geometry. No significant influence in the $T_{C}^{*}$ determination was detected when we changed the *J_int_* between the Co and Gd interface, i.e., by adjusting the *J_int_* value for the Co@Gd core–shell system with total diameters of 2 and 4 nm. Then, we can conclude that the compensation temperature is not exclusive for the *J_int_* sign, since it is seen in all *M(T)* curves for all tested sizes. Indeed, it seems to depend on the fraction of FM structures and also the thermal effects on the individual magnetizations in Co@Gd core–shell structures. Further simulations with the presence of an external field will be carried out to better interpret this issue. On the other hand, it was shown that there is a strong dependence on the sign of *J_int_* for the shape of the hysteresis curves and for the extracted magnetic parameters. We demonstrated that the sign of the magnetic interfacial exchange constant is an important parameter in bi-magnetic core–shell systems that may lead the nanohybrid materials to an exchange spring behavior or not (*J_int_* > 0 yields an enhancement of the coercive field, for *J_int_* < 0 the enhancement is more pronounced, and in addition the core–shell behaves as a magnetic spring). Therefore, on the one hand, it is shown that the interfacial exchange energy is the key to improving the magnetic response for a desired application where a critical temperature can be tuned, adjusting the sizes of core and shell contributions. On the other hand, all the theoretical results presented in this work have to be checked experimentally to fully demonstrate their impact in future biomedical applications.

## Data Availability

The simulated data of the present research can be provided upon reasonable request at the email juan.ramos5@unmsm.edu.pe.

## Supplementary Materials

The following are available online at https://www.mdpi.com/article/10.3390/nano11061381/s1, Figure S1: Normalized *M(T)/M_s_* curves for the core–shell bi-magnetic Co@Gd NP with total diameter of 2, 4, and 6 nm (a) for a positive interfacial exchange. Dependence of the $T_{C}^{*}$ on the Co core size for a total particle size: 2 nm (b), 4 nm (c), and 6 nm (d). Figure S2. Normalized *M(T)/M_s_* curves for the core–shell bi-magnetic Co@Gd NP with total diameter of 2, 4, and 6 nm (a) for a zero interfacial exchange. Dependence of the $T_{C}^{*}$ on the Co core size for a total particle size: 2 nm (b), 4 nm (c), and 6 nm (d). Figure S3: Normalized *M(H)/M_s_* curves under different Δ*t* values at 0 K (a) and Δ*t* dependence of the H_c_ and *M/M_s_* parameters (b). Figure S4. Normalized *M(H)/M_s_* curves for different Co core size for a total core–shell size of 2 nm (a) and 4 nm (b) for positive interfacial exchange constant. Dependence of the Co core size of the *H_c_* and *M/M_s_* values for 2 nm (c) and 4 nm (d). Figure S5. Normalized *M(H)/M_s_* curves for different Co core size for a total core–shell size of 2 nm (a) and 4 nm (b) for zero interfacial exchange constant. Dependence of the Co core size of the *H_c_* and *M/M_s_* values for 2 nm (c) and 4 nm (d).
