# Supplementary material for: Magnetic Simulations of Core–Shell Ferromagnetic Bi-Magnetic Nanoparticles: The Influence of Antiferromagnetic Interfacial Exchange
Source: Nanomaterials (Basel). 2021 May 24;11(6):1381. doi: 10.3390/nano11061381 (PMC8225053; doi:10.3390/nano11061381)
Supplement: Supplementary file 1 [file nanomaterials-11-01381-s001.zip › nanomaterials-1207012-supplementary.pdf]

Supporting Information for Magnetic Simulations of Core–Shell Ferromagnetic Bi-Magnetic Nanoparticles: The Influence of Antiferromagnetic Interfacial Exchange

Juan A. Ramos-Guivar [1,*], Carlo A. Tamanaha-Vegas [1], Fred Jochen Litterst [2,3] and Edson C. Passamani [4]

[1] Grupo de Investigación de Nanotecnología Aplicada para Biorremediación Ambiental, Energía, Biomedicina y Agricultura (NANOTECH), Facultad de Ciencias Físicas, Universidad Nacional Mayor de San Marcos, Av. Venezuela Cdra 34 S/N, Ciudad Universitaria, Lima 15081, Perú; carlo.levano@unmsm.edu.pe

[2] Institut für Physik der Kondensierten Materie, Technische Universität Braunschweig, 38106 Braunschweig, Germany; j.litterst@tu-braunschweig.de

[3] Centro Brasileiro de Pesquisas Físicas, Rio de Janeiro 22290-180, Brazil

[4] Department of Physics, Federal University of Espirito Santo-UFES, Vitória 29075-910, Brazil; edson.caetano@ufes.br

* Correspondence: juan.ramos5@unmsm.edu.pe; Tel.: +51-1-914728212

Supplementary figures

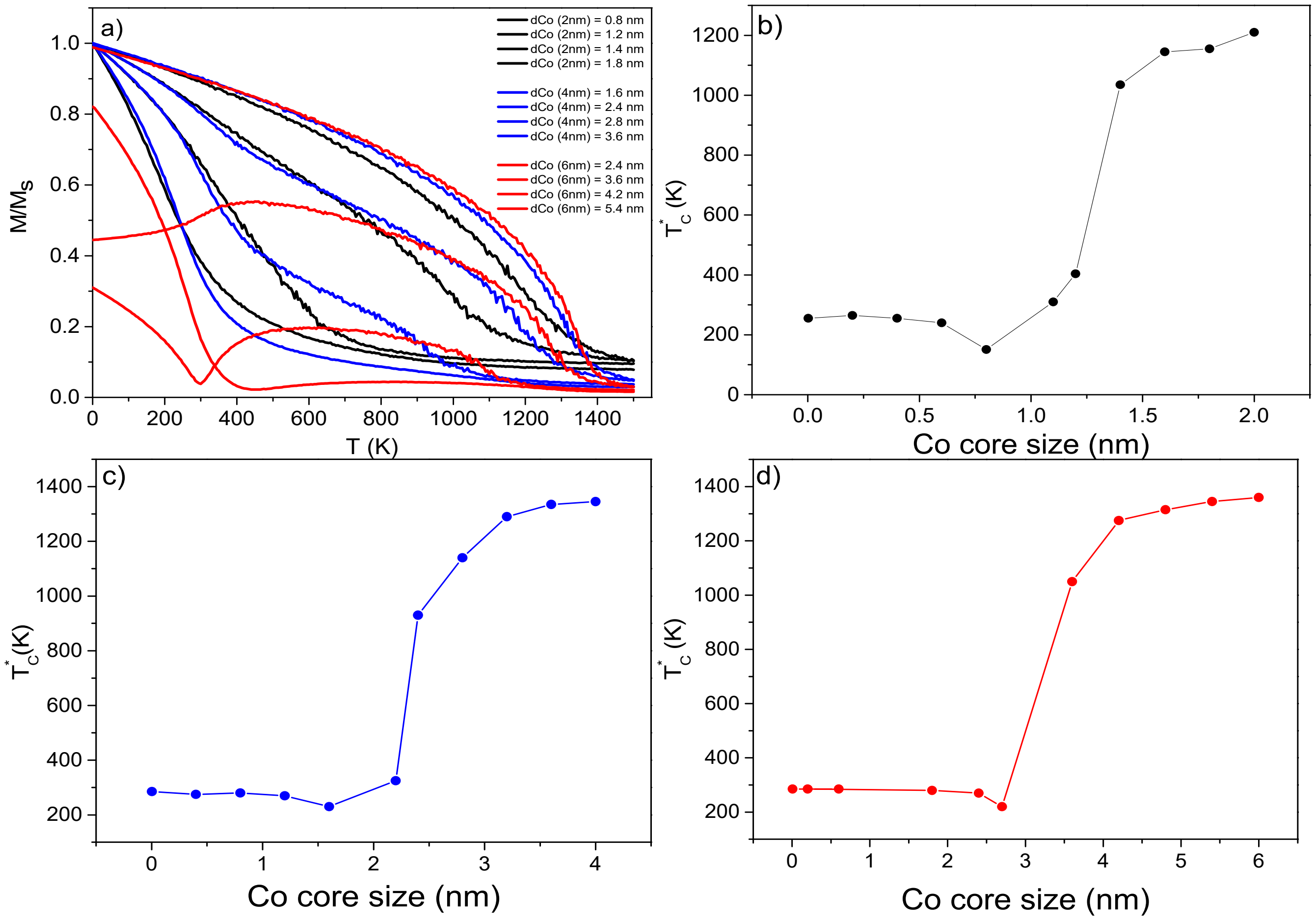

Figure S1. Normalized *M(T)/M_S* curves for the core-shell bi-magnetic Co@Gd NP with total diameter of 2, 4, and 6 nm (a) for a positive interfacial exchange. Dependence of the T_C^* on the Co core size for a total particle size: 2 nm (b), 4 nm (c), and 6 nm (d).

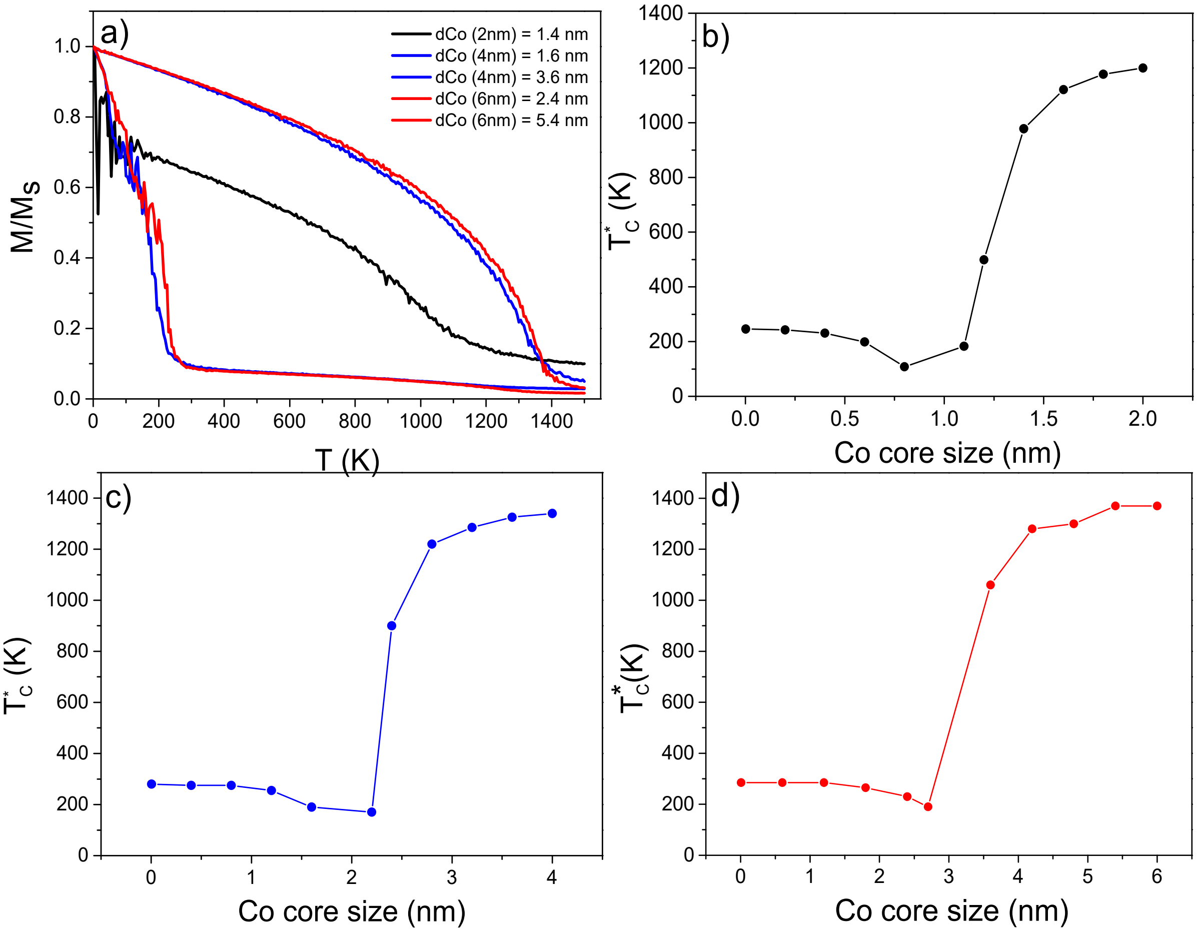

Figure S2. Normalized $M(T)/M_S$ curves for the core-shell bi-magnetic Co@Gd NP with total diameter of 2, 4, and 6 nm (a) for a zero interfacial exchange. Dependence of the T_C^* on the core Co size for a total particle size: 2 nm (b), 4 nm (c), and 6 nm (d).

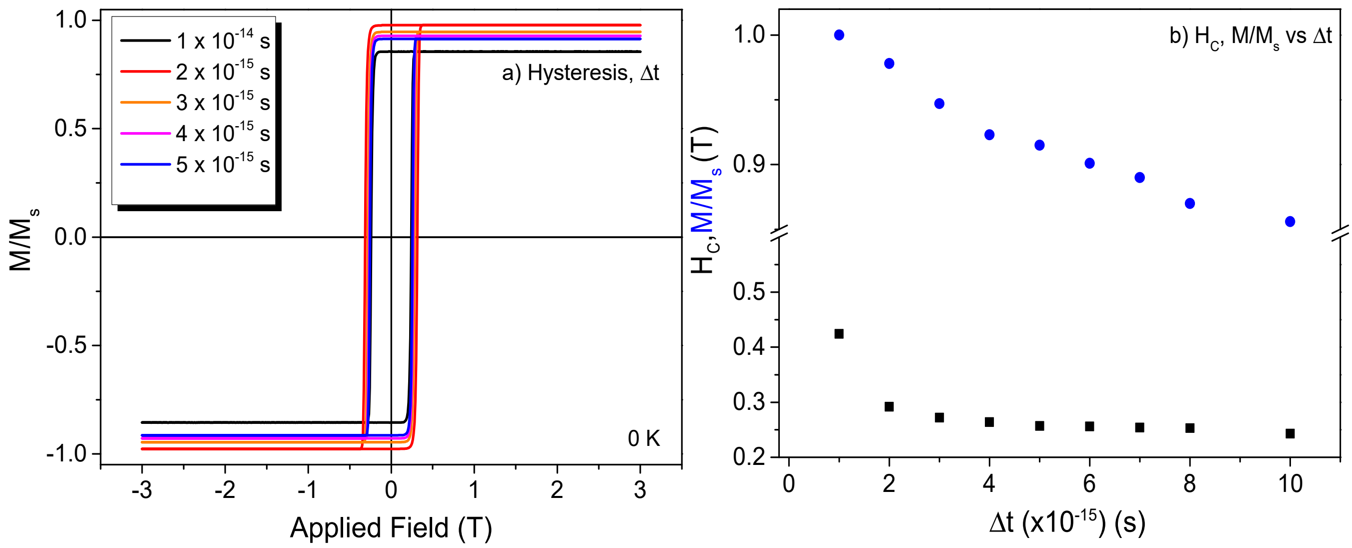

Figure S3. Normalized *M(H)* curves under different *Δt* values at 0 K (a) and *Δt* dependence of the H_c and M/M_s parameters (b).

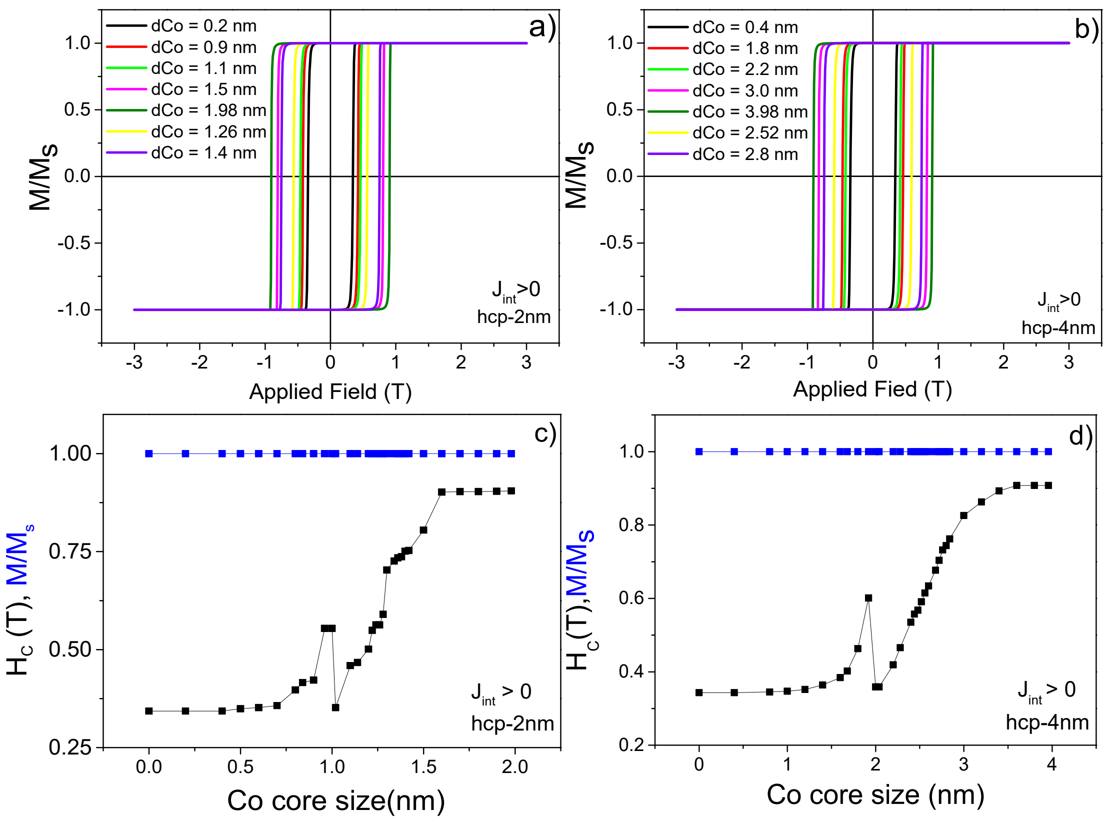

Figure S4. Normalized $M(H)/M_S$ curves for Co core size for a total core-shell size of 2 nm (a) and 4 nm (b) for positive interfacial exchange constant. Dependence of the Co core size of the H_c and M/M_s values for 2 nm (c) and 4 nm (d).

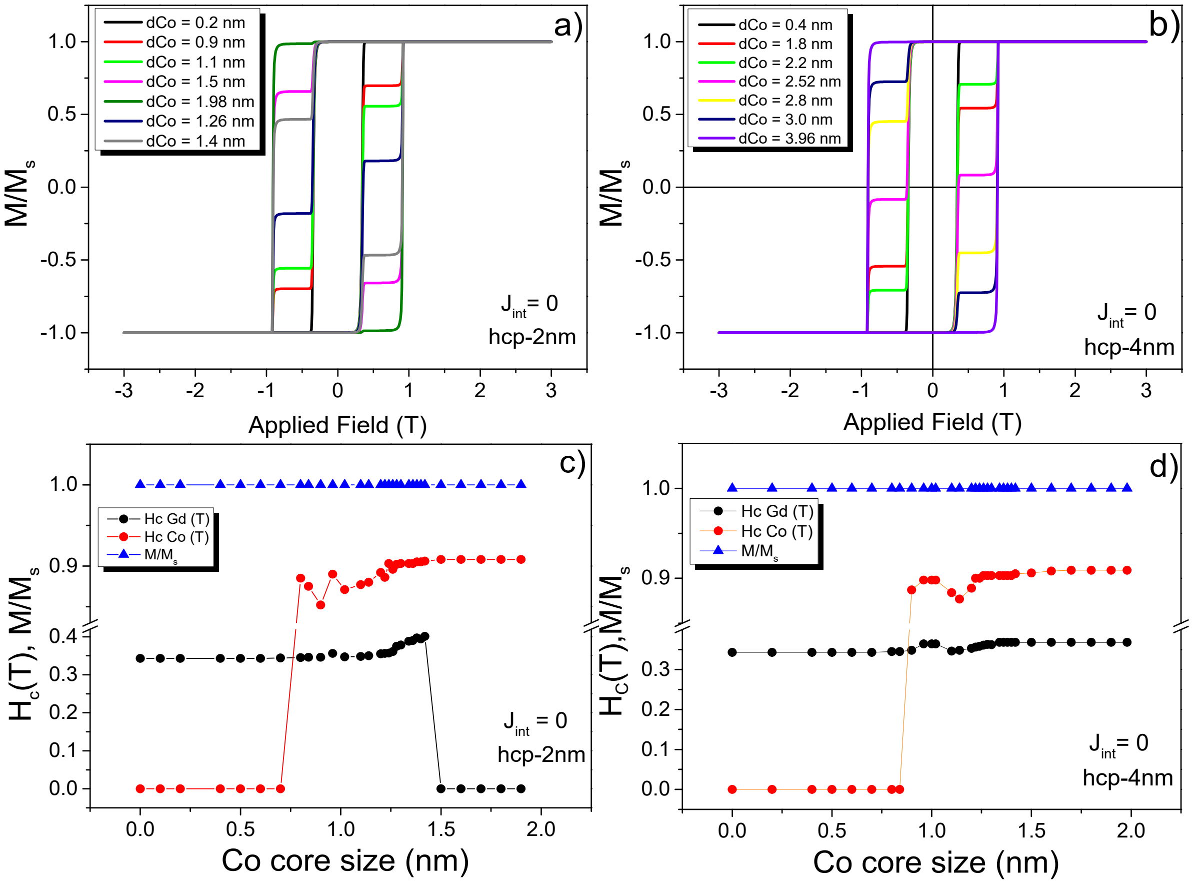

Figure S5. Normalized $M(H)/M_S$ curves for different Co core size for a total core-shell size of 2 nm (a) and 4 nm (b) for zero interfacial exchange constant. Dependence of the Co core size of the H_c and M/M_s values for 2 nm (c) and 4 nm (d).

Supplementary Tables

Table S1. Critical exponents obtained using the finite-size scaling law fitting for Co and Gd NPs. T_c bulk values of Co = 1388 K [2] and Gd equal to 293 K [7] for reference. The errors are found between parenthesis.

	Spheres	Cylinders	Cubes
**T_c-Co (K)**	1388 (2)	1225 (2)	1388 (2)
**d_0-Co**	0.33 (1)	0.30 (3)	0.31 (3)
**z-Co**	1.21 (3)	1.12 (5)	1.14 (6)
**T_c-Gd (K)**	290 (3)	290 (3)	290 (3)
**d_0-Gd**	0.42 (2)	0.37 (2)	0.30 (3)
**z-Gd**	1.37 (5)	1.37 (7)	1.21 (7)
